# The Latest Medical Publications

**Published:** 1861-11

**Authors:** 


THE LATEST MEDICAL PUBLICATIONS.—We have just received, too late for more extended notice, the following recent publications, which we refer to very briefly, as our want of space prevents us from doing them justice in any other way.

1. The Diseases of the Prostate, their Pathology and Treatment; comprising the second edition of "The Enlarged Prostate," and a dissertation on "The Healthy and Morbid Anatomy of the Prostate Gland." Jacksonian Prize Essay for 1860. By Henry Thompson, F.R.C.S., Assistant-Surgeon to University College Hospital, etc. 8vo., pp. 360. London: John Churchill, 1861.

This valuable and handsome work, creditable alike to every one concerned in its publication, and especially to its author, to whom also the Jacksonian prize for 1852 was awarded on "The Pathology and Treatment of Stricture of the Urethra and Urinary Fistulæ," is deserving of commendatory notice, which we should be glad, were it in our power, to give to the fullest extent.

2. On the Parasitic Affections of the Skin. By T. McCall Anderson, M.D., Physician to the Dispensary for Skin Diseases, etc. 8vo., pp. 152. London: John Churchill, 1861.

Interesting from its own merits as a careful analysis and discussion of parasitic cutaneous diseases, and also because of the novelty of the subject. The affections referred to are favus, tinea tonsurans, alopecia areata, pityriasis versicolor, phthiriasis, and scabies.

3. The Causes and Treatment of Imperfect Digestion. By Arthur Leared, M.D., etc. 12mo., pp. 218. London: John Churchill, 1861.

4. On the Sounds caused by the Circulation of the Blood. By the same author. (Pamphlet.) 8vo., pp. 22. London: John Churchill, 1861.

These publications—the latter a thesis for the degree of M.D. in the winter of 1860, the former, we are told, the result of a long-continued attention to derangements of digestion—exhibit accuracy of observation and reflection.

5. Lectures on the Diseases of Women. By Charles West, M.D., F.R.C.P., etc. Second Amer. from the 2d Lond. edition. 8vo., pp. 483. Blanchard & Lea, 1861.

An improved edition of a work which has already established itself in the favor of the profession as a complete treatise on the important subject of which it treats.

